# Nutrient Profiles of Dishes Consumed by the Adequate and High-Salt Groups in the 2014–2018 National Health and Nutrition Survey, Japan

**DOI:** 10.3390/nu13082591

**Published:** 2021-07-28

**Authors:** Hidemi Takimoto, Emiko Okada, Jun Takebayashi, Yuki Tada, Takahiro Yoshizaki, Yuri Yokoyama, Yoshiko Ishimi

**Affiliations:** 1 Department of Nutritional Epidemiology and Shokuiku, National Institute of Health and Nutrition, National Institutes of Biomedical Innovation, Health and Nutrition, 1-23-1 Toyama, Shinjuku-ku, Tokyo 162-8636, Japan; okadae@nibiohn.go.jp; 2 Department of Food Function and Labeling, National Institute of Health and Nutrition, National Institutes of Biomedical Innovation, Health and Nutrition, 1-23-1 Toyama, Shinjuku-ku, Tokyo 162-8636, Japan; jtake@nibiohn.go.jp; 3 Department of Nutritional Science, Faculty of Applied Biosciences, Tokyo University of Agriculture, 1-1-1 Sakuragaoka, Setagaya-ku, Tokyo 156-8502, Japan; y3tada@nodai.ac.jp; 4 Department of Nutrition and Health Sciences, Faculty of Food and Nutritional Sciences, Toyo University, 1-1-1 Izumino, Itakura-cho, Oura-gun, Gunma 374-0193, Japan; yoshizaki@toyo.jp; 5 Research Team for Social Participation and Community Health, Tokyo Metropolitan Institute of Gerontology, 35-2 Sakae-cho, Itabashi-ku, Tokyo 173-0015, Japan; yokoyama@tmig.or.jp; 6 Research Institute for Agricultural and Life Sciences, Tokyo University of Agriculture, 1-1-1 Sakuragaoka, Setagaya-ku, Tokyo 156-8502, Japan; yi207200@nodai.ac.jp

**Keywords:** sodium, dish, dietary survey

## Abstract

Dish-based nutrient profile analyses are essential for setting goals to achieve a balanced diet. In 2014, the Japanese government proposed the “Healthy Meal” criteria, which requires a salt content of 3 g/650 kcal per meal. To examine the current intake status of a nationally representative sample, we conducted a series of secondary analyses of the 2014–2018 National Health and Nutrition Survey data. Participants (aged 18–74 years) were grouped as “high-salt” consumers if their salt intake was 3 g/650 kcal or higher and “adequate” consumers if they consumed less than 3 g/650 kcal. A total of 13,615 participants were identified as “adequate” consumers and 22,300 as “high-salt” consumers. The median salt intake in the “high-salt” group was 11.3 g/day, while that in the “adequate” group was 7.5 g/day. Almost all dishes consumed by the “adequate” group had significantly high energy and fat content but low salt content, compared with those consumed by the “high-salt” group. For example, the median energy, fat, and salt contents in the main dishes consumed by the “adequate” group were 173 kcal, 10.4 g, and 0.9 g/dish, respectively, while those in the main dishes consumed by the “high-salt” group were 159 kcal, 8.9 g, and 1.1 g/dish, respectively. Examples of balanced dishes that are low in both salt and fat content can be proposed to help improve the Japanese consumers’ dietary behavior.

## 1. Introduction

High dietary salt intake persists among the Japanese population; the use of seasonings with high-salt content, such as soy sauce and miso (fermented soybean paste), is essential for preparing traditional Japanese dishes [1]. We have previously reported that people with a high intake of soy sauce and/or miso do not exhibit a higher blood pressure; however, this observation was based on a single one-day food record, and their habitual intake was not assessed [2]. Numerous studies that examined the association between salt intake and disease risk have reported a relationship between high-salt intake and elevated risk of stomach cancer [3], cardiovascular disease [4], and chronic kidney disease [5]. Recommendations aimed at reducing salt intake provided by public health organizations such as the World Health Organization [6] (<5 g/day) and the Japanese government (Dietary Reference Intakes [DRIs], <7.5 g/day for men and <6.5 g/day for women) [7] are currently in place. Furthermore, the guidelines for “Healthy Meals” were proposed by the Japanese government in 2014 [8]. In this report, the committee proposed a standard to reduce the risk of non-communicable diseases (Supplementary Table S1). During the discussion on the criteria for “Healthy Meals”, the committee concluded that special considerations are needed for nutrients whose intake reported in the 2012 National Health and Nutrition Survey, Japan (NHNS-J) exceeded the “tentative dietary goal (DG) for preventing life-style related diseases” stipulated in the 2015 DRIs [9]. For energy intake per day, the mean estimated energy requirement stipulated in the 2015 DRIs, from the eight groups stratified according to sex and age (2194 kcal/day), with medium physical activity levels, was applied. A similar approach was applied to calculate the salt intake. The mean salt DG of the eight groups was also calculated (7.5 g/day). Based on the assumption that 30% of the total energy would be consumed in one meal, the energy content was rounded off to 650 kcal per meal, while salt content was rounded off to 3 g per meal. As a result, the proposed upper limit of the salt content of a meal consisting of staple, main, and side dishes was set at 3 g/650 kcal.

The standard content of the “Healthy Meal” was derived from mathematically optimized values estimated by applying the dietary datasets from the 2012 NHNS-J [10]. Seven years have passed since this standard was made public; hence, there is a need to examine the current intake status of the Japanese adult population by applying nationally representative data. The Japanese enjoy longevity, but their current diet is still high in salt; adults in the 2019 NHNS-J had a mean salt intake of 10.1 g/day [11]. However, studies regarding the nutrient profiles of dishes consumed by the general population, especially those that exceed the current standard, are limited. To identify the actual nutrient profiles of dishes consumed by adequate/high-salt consumers among the nationally representative sample, we conducted a series of secondary analyses of the 2014–2018 National Health and Nutrition Survey data.

## 2. Materials and Methods

Anonymized data from the 2014–2018 NHNS-J were obtained from the Ministry of Health, Labour, and Welfare. The NHNS-J is a nationally representative survey that is conducted annually in November by the Ministry of Health, Labour, and Welfare, Japan. The survey data is exempt from informed consent from the participants, as the survey is implemented based on the “Health Promotion Act.” The survey areas were based on randomly selected census units stratified by prefecture (the Japanese equivalent to a province). All household members residing in the survey area aged 1 year and older were invited to participate in this survey [10,11]. We applied two types of datasets, the dietary record data and the participants’ background data (age, sex, and pregnancy status of women), including the height and weight measurements obtained during the physical examination conducted in the NHNS-J.

### 2.1. Calculation of Dietary Data

The detailed method used to conduct the dietary survey in the NHNS-J has been published previously [12]. In short, a single-day dietary survey record was provided to the participating households, and the members who were in charge of preparing the meals recorded all the names of the dishes consumed, the name of the food item, the weight/portion size of each food item, and the usage and volume of seasonings used, such as soy sauce or salt. When a particular dish was shared among the household members, the approximate proportion of the dish consumed by each member was also recorded so that the individual intake could be calculated. The dietary data for breakfast, lunch, dinner, and snacks were recorded on separate pages. Trained dieticians at the local public health centers in charge of conducting the NHNS-J met the record keepers in person to check the dietary records. After intensive checking by the dieticians, the dietary data were collected using specific software for the NHNS-J.

**Figure 1:**
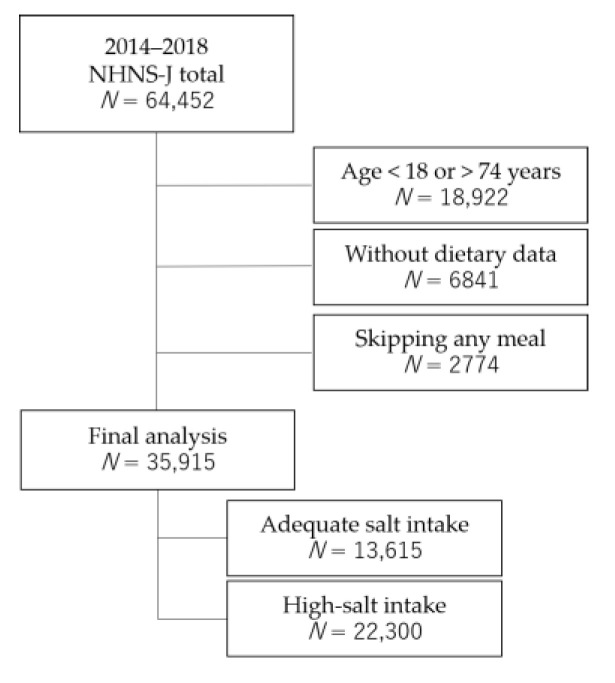
The participant selection process.

The individual dietary record data consisted of dishes and their food items, the method used to cook each food item within the dish (boiling or roasting), and the amount of food item consumed. A “dish” not only includes foods such as soup or boiled rice, but also fruits, confectionaries, or beverages. For example, a cup of coffee, which consists of brewed coffee, milk, and sugar, is counted as one dish. Data regarding the dishes consumed also included information about the type of meal (breakfast, lunch, dinner, or snack). Each food item is accompanied by a unique food number according to the Standard Food Composition Tables in Japan. We applied the 7th Revised version of the Standard Food Composition Tables [13] to calculate the energy and nutrient contents of each dish consumed. We selected the nutrients whose DG values were shown in the 2020 DRIs for Japanese [7]. The proportions of energy (% energy) from protein, fat, and saturated fat were calculated using total energy and energy from each nutrient, according to the following equations. The amount of energy from protein, fat, and saturated fat was calculated as 4 (kcal) × protein (g), 9 (kcal) × fat (g) and saturated fat, by applying Atwater factors. The % energy from carbohydrates was calculated by subtracting the sum of % energy from protein and fat from 100%. The dietary data of 35,915 participants in the 2014–2018 National Health and Nutrition Survey, aged 18–74 years, and who ate three meals on the survey day were used for analyses. Breakfast, lunch, or dinner skipping was defined as no food or drink (excluding water) consumed during mealtime. The participant selection process is illustrated in Figure 1.

We extracted the data of 530,481 dishes consumed on the survey day and categorized them according to the definition of “staple dish” (grain dish), “main dish” (meat, fish, eggs, or beans), and “side dish” (vegetables, seaweeds, potatoes, or mushrooms) shown in the 2005 “Japanese Food Guide Spinning Top” (Supplementary Table S2 [14]). Salt intake (g) was calculated using the following equation: 2.54 × sodium (mg)/1000. Participants were grouped as “adequate” salt consumers if their salt intake was less than 3 g/650 kcal and “high-salt” consumers if they consumed 3 g/650 kcal or more.

### 2.2. Statistical Analysis

Mean, standard deviations (SD), 25th percentile, median, and 75th percentile values were calculated for continuous variables. The adequate and high-salt consumers were compared using the two-sided t-test for normally distributed variables and Wilcoxon’s signed-rank test for skewed variables. For categorical variables, two-sided chi-square tests were used. The nutrient profile of each dish category was also compared between the two groups using Wilcoxon’s signed-rank test. All statistical analyses were conducted using SAS version 9.4 software (SAS Institute Inc. Cary, NC, USA). A *p* value of less than 0.05 (two-sided) by Kruskal–Wallis test for skewed continuous variables was considered significant.

## 3. Results

The general characteristics of the study participants, together with their energy and nutrient intake on the survey day, are shown in Table 1. Of the 35,915 participants selected for the current study, 13,615 were identified as adequate salt consumers and 22,300 as high-salt consumers. Compared with adequate salt consumers, high-salt consumers were significantly older and had a higher proportion of women. Significant differences were observed between the two groups in terms of the intake of energy and most nutrients, except niacin. Adequate salt consumers had significantly higher intake of energy, fat, saturated fat, cholesterol, carbohydrates, % energy from fat, saturated fat and carbohydrates, vitamin E, vitamin B6, pantothenic acid, zinc, and copper compared with high-salt consumers. Salt intake differed between the two groups; the median (25th and 75th percentile values) salt intake was 7.5 g (2.6 and 9.2) and 11.3 g (9.3 and 13.9)/day in the adequate and high-salt consumers, respectively. A significant difference was also observed in the proportion of participants with salt intake within the DGs (7.5 g/day for men and 6.5 g/day for women) shown in the “Dietary Reference Intakes for Japanese, 2020” [7]. Approximately 39.9% of adequate salt consumers had a salt intake within the DGs, while 4.8% of high-salt consumers had a salt intake within the DGs. The intake of foods (g/day) among adequate and high-salt consumers according to the food categories in the 2014–2018 NHNS-J report are shown in Table 2. Adequate salt consumers had a significantly higher intake of cereals and grains, seeds and nuts, fruits, meat, milk and dairy products, fats and oils, confectionaries, and beverages, compared with high-salt consumers. However, adequate salt consumers had a significantly lower intake of sugar and sweeteners, pulses, vegetables, mushrooms, seaweeds, and fish and shellfish.

The distribution of the type of dishes consumed by adequate and high-salt consumers is shown in Table 3. The mean (SD) numbers of dishes consumed on the survey day were 14.62 (4.58) and 14.86 (4.79) in adequate and high-salt consumers, respectively. When all dishes were categorized according to the criteria shown in Supplementary Table S2, approximately one-third of the dishes was “uncategorized,” which suggested that they included foods counted as staple, main, or side dishes but did not fulfill the criteria. The distribution of the types of dishes consumed was similar in both groups. 

The energy and nutrient profiles of each type of dish consumed by adequate and high-salt consumers, excluding dishes without foods counted as staple, main, or side dishes, are shown in Table 4 and Table 5. All types of dishes consumed by adequate salt consumers had significantly higher energy content than those consumed by high-salt consumers. Except for staple dishes, all dishes consumed by adequate salt consumers had significantly higher fat and saturated fat contents and lower sodium (salt) content. Significant differences in the % energy from protein, fat, saturated fat, and carbohydrates were observed between the two groups among all types of dishes, except for the two combined dishes (staple and side, staple and main). Dishes eaten by adequate salt consumers had higher % energy from fat and saturated fat, but lower % energy from protein compared to high-salt consumers, except for staple dish where % energy from fat and saturated fat were lower.

## 4. Discussion

Using the 2014–2018 NHNS-J dietary data from 35,915 participants aged 18–74 years, we were able to compare the characteristics of the dishes consumed by adequate salt (<3 g/650 kcal) and high-salt consumers from a nationally representative sample. By limiting the data to those consuming breakfast, lunch, and dinner on the survey day, we were able to exclude participants with low salt intake due to low total food intake.

High-salt consumers had higher mean age and a higher proportion of women than adequate salt consumers. This finding is in accordance with the reports of previous studies on Japanese adults [1,12,15]. In terms of the absolute amount of salt intake, women exhibit lower intake compared with men, but their energy-adjusted intake is higher [12]. In the 2019 NHNS-J, mean salt intake per 1000 kcal among women aged 20 years and older was 5.5 g but that among men was 5.2 g [11].

The nutrient profiles of the dishes consumed by adequate salt consumers present a unique insight into how a dish could be composed based on the official food-based guidelines. Previous studies have mainly focused on individual food items or types of foods that are dietary sources of sodium [1,15,16], not as familiar dishes. Some previous studies reported on the nutrient composition of selected dishes [17,18], but the nutrient composition of these dishes is based on recipes, rather than the actual intake. As the major source of salt in the Japanese diet is seasonings, which make up 67% of salt intake among Japanese people aged 1 year and older [11], it is particularly important to estimate the salt content of dishes consumed. In this study, we categorized the dishes recorded in the dietary survey data according to the “Healthy Meal” criteria [8]. The current data could be used as an example of an adequate salt dish. Furthermore, we were able to present the nutrient profiles of dishes with small amounts of food counted as staple, main, and side dishes, which comprised a significant proportion (nearly one-third) of the total number of dishes consumed on the survey day. More than 25% of these small-sized dishes consumed by high-salt consumers had a salt content greater than 1.0 g/dish (Table 5). Reducing the amount of salt in small-sized dishes may be practical advice to follow.

The protein content of dishes consumed by adequate salt consumers was similar to that consumed by high-salt consumers, but the fat and saturated fat contents were significantly higher, except those in staple dishes. This was probably because adequate salt consumers had higher intake of milk and dairy products, and fats and oils (Table 2). The differences in the intake of food groups among the adequate and high-salt consumers in this study are similar to those of our previous analyses using the 2012 NHNS-J data [19]. In this study, a Westernized diet low in sodium was also characterized by higher intake of milk and dairy products, butter, and margarine, but lower intake of miso (fermented soybean paste). On the contrary, the traditional Japanese diet high in sodium was characterized by higher intake of potatoes, sugar and sweeteners, vegetables, mushrooms, fish, shellfish, soy sauce, and miso. The adequate salt consumers in our study may have consumed a more Westernized diet compared with the high-salt consumers; however, the results in Table 4 show that side dishes consisting of vegetables, potatoes, pulses (excluding soy), seaweeds, or mushrooms consumed by the adequate salt consumers have similar amounts of dietary fiber and slightly less potassium. Likewise, the combined dishes including side dishes (Table 5) consumed by adequate salt consumers have similar amounts of dietary fiber and potassium compared with those consumed by high-salt consumers. These results show that it is possible to prepare dishes from plant sources without adding excess Japanese traditional seasonings. The macronutrient composition of different kinds of “dishes” is also valuable in creating healthier recipes for packaged or take-away meals. As most types of dishes eaten by adequate salt consumers had higher % energy from fat or saturated fat compared to high-salt consumers (Table 4 and Table 5), there is a need to develop recipes that are low in sodium as well as moderate in fat. This is especially important, as nearly half of the study subjects exceeded the DG (7% of energy) for saturated fat shown in the DRIs [7].

The current study has some limitations. Information on where these dishes were prepared was unavailable. In the NHNS-J, this information (home-prepared, take-aways, eating out, etc.) is collected for each meal, not for each dish. In the 2019 NHNS-J, the proportion of adults aged 20 years and older who never eat out was 22.2%, while the proportion of those who never eat take-away was 23.0% [11], which means that most Japanese adults have chances to eat out or eat take-aways at home. We cannot rule out the possibility that the dishes consumed by adequate salt consumers were prepared away from home, thereby considering the possibility that these dishes had higher fat and saturated fat contents. Additionally, we cannot completely rule out the possibility that there was underreporting of seasonings added at the table, though the dietary records were checked by trained dieticians. According to a recent review [20], assessment of sodium intake by dietary records may be influenced by behavioral alterations of the survey participants, such as selecting healthier foods on the survey day, leading to underestimation of usual intakes. We need to take these limitations into account when applying the current study results to the general population.

However, the current results present valuable examples of how dishes with adequate sodium content can be prepared. East Asian countries such as China share a similar problem with high-sodium diets [21] as in Japan. We hope that our results could be utilized to propose balanced dishes low in both salt and fat to help modify the diet of the population.

## Figures and Tables

**Table 1:** General characteristics and energy and nutrient intake per day of adequate and high-salt consumers aged 18–74 years in the 2014–2018 National Health and Nutrition Survey, Japan.

	Adequate						High						
	*N*	Mean	SD	25th Percentile	Median	75th Percentile	*N*	Mean	SD	25th Percentile	Median	75th Percentile	*p*-Value
Age (years)	13,615	50.52	15.34	39	51	64	22,300	53.27	15.06	42	56	66	<0.01
Height (cm)	10,924	162.25	9.06	155.5	162	169	17,896	160.67	9.01	154	160	167.2	<0.01
Weight * (kg)	10,845	60.39	12.29	51.3	59	68	17,769	59.96	12.14	51	58.2	67.3	<0.01
BMI ^†^	10,844	22.83	3.62	20.28	22.35	24.82	17,764	23.12	3.65	20.53	22.69	25.15	<0.01
Women (N, %)	6811	50%					12,680	57%					<0.01
Energy (kcal)	13,615	2081	591	1681	2014	2408	22,300	1901	535	1537	1845	2201	<0.01
Protein (g)		73.1	23.9	57.0	70.6	86.5		73.7	23.6	57.5	71.2	86.7	0.03
Fat (g)		65.1	27.0	46.5	61.8	79.6		58.3	23.8	41.8	55.2	71.3	<0.01
Saturated fat (g)		19.2	9.4	12.8	17.8	24.0		16.7	8.0	11.1	15.5	21.0	<0.01
Cholesterol (mg)		336	192	191	315	440		329	192	185	307	433	<0.01
Carbohydrates (g)		275	85	218	267	321		252	76	200	246	295	<0.01
Dietary fiber (g)		19.1	7.2	14.3	18.2	23.0		19.6	7.2	14.7	18.8	23.5	<0.01
Vitamin A (μgRAE)		538	813	253	399	609		556	1004	261	408	618	0.01
Vitamin D (μg)		6.6	7.9	1.6	3.3	9.2		7.6	8.8	1.7	4.1	10.8	<0.01
Vitamin E (mg)		7.0	3.5	4.6	6.4	8.7		6.8	3.3	4.6	6.3	8.5	0.02
Vitamin K (μg)		241	177	110	189	336		255	187	117	204	352	<0.01
Vitamin B_1_ (mg)		0.96	0.46	0.65	0.87	1.18		0.97	0.46	0.66	0.88	1.19	0.01
Vitamin B_2_ (mg)		1.17	0.50	0.85	1.11	1.42		1.19	0.51	0.85	1.12	1.44	0.03
Niacin (mgNE)		31.8	11.8	23.7	30.1	37.9		31.7	11.7	23.7	30.1	37.7	0.67
Vitamin B_6_ (mg)		1.23	0.52	0.86	1.15	1.51		1.21	0.50	0.86	1.14	1.48	<0.01
Vitamin B_12_ (μg)		5.87	6.11	2.03	3.82	7.40		6.81	7.17	2.26	4.55	8.73	<0.01
Folate (μg)		290	144	197	269	353		309	156	211	285	376	<0.01
Pantothenic acid (mg)		5.9	2.1	4.5	5.7	7.0		5.6	2.0	4.2	5.4	6.7	<0.01
Vitamin C (mg)		98.1	76.3	46.0	78.0	128.4		101.7	72.3	49.9	84.1	134.7	<0.01
Sodium (mg)		3038	1003	2364	2950	3613		4663	1464	3654	4456	5456	<0.01
Potassium (mg)		2336	908	1713	2234	2835		2408	892	1778	2297	2901	<0.01
Calcium (mg)		489	280	304	444	623		512	250	329	469	647	<0.01
Magnesium (mg)		252	94	187	239	302		265	95	199	252	317	<0.01
Phosphorus (mg)		1017	347	787	983	1209		1028	336	796	994	1218	<0.01
Iron (mg)		7.6	3.0	5.6	7.2	9.2		8.2	3.1	6.1	7.8	9.9	<0.01
Zinc (mg)		8.8	3.2	6.7	8.4	10.4		8.3	3.0	6.3	7.9	9.8	<0.01
Copper (mg)		1.19	0.44	0.90	1.14	1.42		1.17	0.41	0.89	1.13	1.39	<0.01
% Energy from protein		14.1	2.8	12.2	14.0	15.9		15.6	3.1	13.6	15.4	17.4	<0.01
% Energy from fat		28.0	7.6	22.9	27.9	32.9		27.4	7.3	22.5	27.2	32.2	<0.01
% Energy from saturated fat		8.3	3.0	6.1	8.0	10.0		7.8	2.8	5.8	7.6	9.6	<0.01
% Energy from Carbohydrates		57.9	8.6	52.3	57.8	63.4		57.0	8.4	51.6	57.1	62.6	<0.01
Salt (g)		7.7	2.6	6.0	7.5	9.2		11.8	3.7	9.3	11.3	13.9	<0.01
Salt intake within DG (N, %)	5438	39.9%					1074	4.8%					<0.01

* Excluding 166 pregnant women; ^†^ participants with both height and weight data after excluding 166 pregnant women; salt (g) = 2.54 × Na (mg)/1000; SD: standard deviations; DG: dietary goals for the prevention and progression of life-style related disease, 7.5 g/day for men and 6.5 g/day for women.

**Table 2:** Intake of foods (g/day) among adequate and high-salt consumers aged 18–74 years in the 2014–2018 National Health and Nutrition Survey, Japan.

	Adequate *n* = 13,615					High *n* = 22,300					
	Mean	SD	25th Percentile	Median	75th Percentile	Mean	SD	25th Percentile	Median	75th Percentile	*p*-Value
Cereals and grains	464.1	196.3	330.0	445.1	571.7	426.0	170.7	308.0	412.0	524.4	<0.01
Potatoes	52.8	69.5	0.0	31.0	78.3	54.8	68.8	0.0	33.8	83.3	0.06
Sugar and sweeteners	6.4	8.6	0.2	3.6	9.0	6.8	8.4	0.8	4.1	9.5	<0.01
Pulses	62.3	81.8	0.0	40.0	90.0	66.5	77.6	3.8	45.0	100.0	<0.01
Seeds and nuts	3.1	10.8	0.0	0.0	1.0	2.3	8.0	0.0	0.0	1.0	<0.01
Vegetables	264.6	165.8	145.7	236.0	352.0	302.5	173.8	177.6	276.1	395.1	<0.01
Fruits	103.5	138.6	0.0	59.5	164.0	96.6	122.6	0.0	58.5	154.8	0.03
Mushrooms	15.7	27.3	0.0	0.0	21.7	19.0	29.6	0.0	6.0	27.8	<0.01
Seaweeds	8.5	20.9	0.0	0.3	9.1	13.2	26.1	0.0	2.3	15.8	<0.01
Fish and shellfish	61.7	65.4	3.0	46.5	97.0	77.3	72.7	15.0	66.0	115.0	<0.01
Meat	109.5	84.6	50.0	96.7	151.0	93.8	75.6	40.0	80.0	132.5	<0.01
Eggs	38.7	36.8	3.6	36.7	57.0	38.7	36.6	2.7	39.6	57.0	0.51
Milk and dairy products	124.9	150.6	0.0	80.0	206.0	99.2	123.2	0.0	51.5	171.0	<0.01
Fats and oils	12.7	10.7	4.9	10.2	18.0	10.8	9.5	4.0	9.0	15.4	<0.01
Confectionaries	31.2	52.3	0.0	0.0	47.0	21.3	39.8	0.0	0.0	30.0	<0.01
Beverages	762.8	559.3	380.0	674.0	1038.0	698.1	491.3	355.0	610.0	950.0	<0.01
Seasonings and spices	49.0	29.4	29.6	44.3	61.8	72.5	43.4	46.0	64.0	88.0	<0.01

SD: standard deviations.

**Table 3:** Distribution of the type of dishes among adequate and high-salt consumers aged 18–74 years in the 2014–2018 National Health and Nutrition Survey, Japan.

Number of Dishes		Adequate		High	
		199,001		331,480	
		*N*	%	*N*	%
Dishes Including Foods Counted as Staple, Main, or Side Dishes	Total number of dishes	126,653	63.64	220,427	66.50
	Staple dish	22,528	11.32	34,173	10.31
	Main dish	16,896	8.49	27,614	8.33
	Side dish	14,980	7.53	28,888	8.71
	Combined dish ^†^ (staple, main, and side)	1747	0.88	2733	0.82
	Combined dish ^†^ (staple and side)	768	0.39	2038	0.61
	Combined dish ^†^ (main and side)	5693	2.86	9832	2.97
	Combined dish ^†^ (staple and main)	1919	0.96	3402	1.03
	Uncategorized dish *	62,122	31.22	111,747	33.71
Miscellaneous	Total number of dishes	72,348	36.36	111,053	33.50
	Fresh fruit and fruit juice	11,218	5.64	18,231	5.50
	Seeds and nuts	623	0.31	766	0.23
	Milk and dairy products	8261	4.15	11,751	3.55
	Pastries and confectionaries	9063	4.55	11,512	3.47
	Beverages	34,527	17.35	55,778	16.83
	Others ^†^	8656	4.35	13,015	3.93

* Includes foods counted as staple, main, or side dishes; ^†^ combinations of two or more foods in this category.

**Table 4:** Comparison of nutrient profiles of staple, main, and side dishes among adequate and high-salt consumers.

	Adequate					High					
	Mean	SD	25th Percentile	Median	75th Percentile	Mean	SD	25th Percentile	Median	75th Percentile	*p*-Value
**Staple Dish**	**Number of Dishes = 22,528**					**Number of Dishes = 34,173**					
Energy (kcal)	347	124	252	336	386	326	105	252	316	363	<0.01
Protein (g)	6.3	3.4	4.0	5.0	7.5	6.7	4.2	3.8	5.0	8.1	0.31
Fat (g)	2.2	4.7	0.5	0.6	1.0	2.9	5.4	0.5	0.6	1.8	0.49
Saturated fat (g)	0.75	1.85	0.16	0.20	0.30	0.97	2.07	0.15	0.20	0.41	0.06
Carbohydrates (g)	72.3	26.5	55.7	70.1	76.7	65.6	20.3	54.0	61.0	74.2	<0.01
Dietary fiber (g)	3.1	1.3	2.3	3.0	3.5	2.9	1.2	2.3	2.7	3.2	<0.01
Sodium (mg)	154	345	2	2	5	381	722	2	2	515	<0.01
Potassium (mg)	84	69	46	58	87	98	92	44	58	111	0.03
Salt (g)	0.4	0.9	0.0	0.0	0.0	1.0	1.8	0.0	0.0	1.3	<0.01
% Energy from protein	7.2	2.7	6.0	6.0	6.4	8.0	3.5	6.0	6.0	9.1	<0.01
% Energy from fat	5.2	9.3	1.6	1.6	2.1	6.9	10.7	1.6	1.6	5.5	<0.01
% Energy from Saturated fat	1.7	3.6	0.5	0.5	0.6	2.3	4.1	0.5	0.5	1.1	<0.01
% Energy from carbohydrates	87.6	11.2	91.2	92.4	92.4	85.1	13.0	82.9	92.4	92.4	<0.01
Total amount of dish (g)	201.7	76.3	150.0	200.0	225.0	192.6	71.1	150.0	180.0	213.5	<0.01
**Main Dish**	**Number of Dishes = 16,896**					**Number of Dishes = 27,614**					
Energy (kcal)	209	137	114	173	261	189	113	109	159	239	<0.01
Protein (g)	15.0	8.6	8.8	13.0	18.4	14.7	7.9	8.8	13.0	18.0	0.12
Fat (g)	13.2	10.7	6.1	10.4	17.3	11.2	8.5	5.5	8.9	15.0	<0.01
Saturated fat (g)	3.27	3.44	1.31	2.25	4.06	2.71	2.62	1.08	1.90	3.45	<0.01
Carbohydrates (g)	5.8	8.0	0.6	3.5	7.4	5.8	8.0	0.5	3.3	7.4	<0.01
Dietary fiber (g)	0.7	1.1	0.0	0.1	0.8	0.6	1.2	0.0	0.1	0.8	<0.01
Sodium (mg)	409	293	198	358	551	525	424	245	432	672	<0.01
Potassium (mg)	261	162	150	231	341	261	155	154	237	340	0.22
Salt (g)	1.0	0.7	0.5	0.9	1.4	1.3	1.1	0.6	1.1	1.7	<0.01
% Energy from protein	31.9	13.3	23.2	30.0	35.1	34.3	14.6	24.8	31.4	39.4	<0.01
% Energy from fat	53.6	15.3	46.1	56.2	64.2	50.2	16.8	42.7	52.1	62.0	<0.01
% Energy from saturated fat	12.7	6.2	8.0	12.4	15.8	11.7	6.1	7.1	11.7	15.1	<0.01
% Energy from carbohydrates	14.5	10.8	6.1	11.3	19.2	15.5	11.9	6.0	11.7	21.6	<0.01
Total amount of dish (g)	118.0	66.1	70.0	100.0	149.5	114.5	61.6	69.3	100.0	141.9	<0.01
**Side Dish**	**Number of Dishes = 14,980**					**Number of Dishes = 28,888**					
Energy (kcal)	114	96	47	88	149	104	84	45	82	136	<0.01
Protein (g)	4.0	3.4	1.5	2.8	5.7	4.1	3.3	1.6	3.0	5.8	<0.01
Fat (g)	4.9	5.9	0.3	2.9	7.4	4.3	5.4	0.3	2.4	6.2	<0.01
Saturated fat (g)	1.07	1.63	0.04	0.42	1.36	0.94	1.54	0.04	0.34	1.14	<0.01
Carbohydrates (g)	13.9	13.6	5.8	9.7	17.1	13.0	11.1	5.9	9.6	16.6	<0.01
Dietary fiber (g)	2.9	1.8	1.7	2.5	3.6	2.8	1.8	1.7	2.5	3.5	0.96
Sodium (mg)	348	323	100	262	515	509	453	174	405	732	<0.01
Potassium (mg)	345	210	213	301	420	339	208	211	297	414	<0.01
Salt (g)	0.9	0.8	0.3	0.7	1.3	1.3	1.2	0.4	1.0	1.9	<0.01
% Energy from protein	16.7	10.0	9.2	14.9	22.0	18.0	10.1	10.8	16.5	23.6	<0.01
% Energy from fat	31.9	23.9	6.6	32.4	51.6	29.6	23.2	6.0	28.2	48.3	<0.01
% Energy from saturated fat	6.2	6.2	0.9	4.9	8.9	5.7	6.1	0.9	4.2	8.2	<0.01
% Energy from carbohydrates	51.5	24.7	32.1	47.1	73.6	52.4	23.7	33.4	49.4	73.7	<0.01
Total amount (g)	155.2	74.1	104.3	136.9	187.5	153.6	69.5	104.0	135.1	185.0	0.11

**Table 5:** Comparison of nutrient profiles of combined * and uncategorized ^†^ dishes among adequate and high-salt consumers.

	Adequate					High					
	Mean	SD	25th Percentile	Median	75th Percentile	Mean	SD	25th Percentile	Median	75th Percentile	*p*-Value
**Staple, Main, and Side**	**Number of Dishes = 1747**					**Number of Dishes = 2733**					
Energy (kcal)	727	234	573	705	851	679	222	529	658	794	<0.01
Protein (g)	27.3	10.5	20.7	25.0	30.0	26.8	10.4	20.0	24.4	30.0	<0.01
Fat (g)	25.9	13.6	15.9	24.8	31.4	23.6	12.6	14.5	22.2	29.5	<0.01
Saturated fat (g)	7.55	4.89	4.20	6.83	9.48	6.95	4.63	3.65	6.16	8.81	<0.01
Carbohydrates (g)	91.0	35.9	66.7	88.1	108.2	85.6	32.1	63.9	82.0	102.4	<0.01
Dietary fiber (g)	7.2	3.0	5.3	6.7	8.7	7.0	3.0	5.0	6.3	8.6	<0.01
Sodium (mg)	1209	528	811	1199	1534	1679	868	1176	1529	2021	<0.01
Potassium (mg)	662	285	487	619	723	652	280	467	606	736	0.09
Salt (g)	3.1	1.3	2.1	3.0	3.9	4.3	2.2	3.0	3.9	5.1	<0.01
% Energy from protein	7.2	2.7	6.0	6.0	6.4	8.0	3.5	6.0	6.0	9.1	<0.01
% Energy from fat	5.2	9.3	1.6	1.6	2.1	6.9	10.7	1.6	1.6	5.5	<0.01
% Energy from saturated fat	1.7	3.6	0.5	0.5	0.6	2.3	4.1	0.5	0.5	1.1	<0.01
% Energy from carbohydrates	87.6	11.2	91.2	92.4	92.4	85.1	13.0	82.9	92.4	92.4	<0.01
Total amount (g)	515.5	168.7	414.9	478.5	561.0	509.4	173.7	402.5	465.4	564.5	0.01
**Staple and Side**	**Number of Dishes = 768**					**Number of Dishes = 2038**					
Energy (kcal)	496	169	380	475	592	464	151	361	460	551	<0.01
Protein (g)	14.0	4.7	10.9	13.9	16.6	14.4	4.8	11.0	14.2	17.1	0.11
Fat (g)	13.2	8.1	7.2	13.1	18.2	12.0	7.9	5.6	11.6	16.7	<0.01
Saturated fat (g)	3.72	2.78	1.43	3.45	5.30	3.43	2.90	1.16	2.85	4.83	<0.01
Carbohydrates (g)	77.7	29.4	58.6	71.7	93.4	72.3	24.1	57.7	69.7	84.1	<0.01
Dietary fiber (g)	6.1	2.2	4.4	5.8	7.2	6.0	2.4	4.4	5.5	7.1	0.08
Sodium (mg)	949	465	620	883	1207	1468	794	923	1350	1838	<0.01
Potassium (mg)	405	172	291	381	490	401	167	292	368	482	0.32
Salt (g)	2.4	1.2	1.6	2.2	3.1	3.7	2.0	2.3	3.4	4.7	<0.01
% Energy from protein	11.8	3.4	9.2	11.3	13.9	12.8	3.3	10.4	12.7	14.9	<0.01
% Energy from fat	23.2	10.5	15.9	23.9	30.3	22.0	10.9	13.9	22.6	29.3	<0.01
% Energy from saturated fat	6.5	3.9	3.3	6.6	8.7	6.1	4.2	2.7	5.9	8.4	<0.01
% Energy from Carbohydrates	65.0	10.8	58.5	64.4	71.8	65.2	10.7	58.3	64.3	72.6	0.58
Total amount (g)	366.2	110.6	293.8	350.0	410.4	365.1	109.3	289.9	352.2	416.0	0.90
**Main and Side**	**Number of Dishes = 5693**					**Number of Dishes = 9832**					
Energy (kcal)	367	201	229	325	458	338	172	214	300	423	<0.01
Protein (g)	21.4	11.7	13.6	18.5	25.6	21.0	11.0	13.4	18.3	25.3	0.15
Fat (g)	21.4	15.1	11.2	18.4	27.6	17.9	12.2	9.1	15.4	24.0	<0.01
Saturated fat (g)	6.55	5.52	2.73	5.19	8.69	5.29	4.52	2.01	3.91	7.30	<0.01
Carbohydrates (g)	20.6	15.3	9.7	16.1	26.8	21.7	15.4	10.7	17.7	28.6	<0.01
Dietary fiber (g)	4.4	2.9	2.3	3.7	5.6	4.4	2.9	2.3	3.7	5.7	0.98
Sodium (mg)	821	514	455	726	1085	1189	769	644	1030	1535	<0.01
Potassium (mg)	632	320	412	558	781	632	322	406	556	775	0.78
Salt (g)	2.1	1.3	1.2	1.8	2.8	3.0	2.0	1.6	2.6	3.9	<0.01
% Energy from protein	25.3	9.7	18.0	24.2	30.0	26.8	9.9	19.8	26.2	31.8	<0.01
% Energy from fat	50.1	14.4	41.9	51.3	59.9	45.4	15.5	35.8	46.8	56.6	<0.01
% Energy from saturated fat	14.8	6.5	9.7	14.5	19.5	12.9	6.6	7.6	12.2	17.5	<0.01
% Energy from carbohydrates	24.6	12.3	14.9	22.2	32.2	27.7	13.1	17.5	25.7	35.7	<0.01
Total amount (g)	333.6	169.6	220.0	287.7	396.9	336.2	163.3	220.7	292.7	402.5	0.08
**Staple and Main**	**Number of Dishes = 1919**					**Number of Dishes = 3402**					
Energy (kcal)	606	184	474	597	711	556	173	437	534	671	<0.01
Protein (g)	22.3	7.8	16.9	21.0	26.7	22.4	7.8	16.9	21.2	26.2	0.69
Fat (g)	18.5	10.3	10.3	18.1	25.2	16.1	9.9	8.1	14.1	22.0	<0.01
Saturated fat (g)	4.97	3.53	2.21	3.91	7.56	4.45	3.46	1.87	3.31	6.61	<0.01
Carbohydrates (g)	82.2	28.5	59.8	83.3	100.5	75.5	25.9	57.5	73.2	91.4	<0.01
Dietary fiber (g)	4.0	1.4	3.0	4.0	4.6	3.9	1.7	2.7	3.5	4.5	<0.01
Sodium (mg)	930	435	594	930	1160	1348	759	840	1172	1656	<0.01
Potassium (mg)	360	163	263	327	436	358	153	261	334	425	0.72
Salt (g)	2.4	1.1	1.5	2.4	2.9	3.4	1.9	2.1	3.0	4.2	<0.01
% Energy from protein	15.2	4.2	12.2	15.0	17.3	16.6	4.3	13.6	16.3	19.3	<0.01
% Energy from fat	26.9	11.3	18.1	26.5	33.7	25.3	11.5	16.2	24.7	32.5	<0.01
% Energy from saturated fat	7.2	4.1	4.0	6.2	9.8	6.9	4.3	3.7	5.4	9.8	<0.01
% Energy from carbohydrates	57.9	11.1	51.1	58.8	65.5	58.1	10.8	51.0	59.8	66.0	0.23
Total amount (g)	348.0	100.2	274.0	350.7	397.6	338.2	98.3	270.0	331.5	396.5	<0.01
**Uncategorized Dish**	**Number of Dishes = 62,122**					**Number of Dishes = 111,747**					
Energy (kcal)	80	75	24	59	116	76	71	22	56	109	<0.01
Protein (g)	3.3	2.8	1.0	2.6	5.3	3.3	2.8	1.0	2.5	5.1	<0.01
Fat (g)	3.2	3.9	0.3	2.1	4.7	2.8	3.5	0.2	1.5	4.2	<0.01
Saturated fat (g)	0.86	1.36	0.07	0.34	1.10	0.72	1.20	0.05	0.24	0.86	<0.01
Carbohydrates (g)	9.3	12.5	1.4	3.5	10.1	9.4	12.3	1.6	3.8	10.2	<0.01
Dietary fiber (g)	0.9	0.8	0.3	0.8	1.5	1.0	0.8	0.4	0.9	1.5	<0.01
Sodium (mg)	222	219	61	167	312	297	311	80	203	412	<0.01
Potassium (mg)	98	86	41	72	131	101	87	41	77	135	<0.01
Salt (g)	0.6	0.6	0.2	0.4	0.8	0.8	0.8	0.2	0.5	1.0	<0.01
% Energy from protein	21.6	14.3	11.0	18.8	30.0	22.1	14.5	10.9	20.0	30.5	<0.01
% Energy from fat	33.8	24.6	7.8	35.0	53.8	30.2	24.0	5.6	28.4	49.3	<0.01
% Energy from saturated fat	7.5	7.2	1.2	6.1	11.1	6.5	6.8	0.8	5.2	9.5	<0.01
% Energy from carbohydrates	44.5	28.5	20.4	40.1	69.4	47.6	28.5	24.1	44.9	71.7	<0.01
Total amount (g)	54.3	45.5	27.4	47.0	72.0	55.3	38.5	29.0	49.0	74.7	<0.01

* Dishes with combinations of foods counted as staple, main, or side dishes. ^†^ Dishes including foods counted as staple, main, or side dishes, but with less than one serving. SD: standard deviations.

## Data Availability

Restrictions apply to the availability of these data. Data was obtained from the Ministry of Health, Labour and Welfare (MHLW), on behalf of the government of Japan. MHLW approves data usage to domestic researchers only, after they have applied according to the guidelines of the national data use. The application form should be written in Japanese, as shown below in https://www.mhlw.go.jp/english/database/anonymized_data/. In the answer to Q.8 of the corresponding webpage, it is stated that some of the government statistical data is available to overseas researchers. However, this applies only to the “Comprehensive Survey of Living Conditions” (https://www.mhlw.go.jp/english/database/anonymized_data/#h4-09). Therefore, the “National Health and Nutrition Survey” data is not permitted for research use to researchers overseas.

## Supplementary Materials

The following are available online at https://www.mdpi.com/article/10.3390/nu13082591/s1, Table S1: Standards for the “Healthy Meal”, Table S2: Criteria for categorizing dishes according to the definitions in the Japanese Food Guide Spinning Top.
