# Supplementary material for: Nutrient Profiles of Dishes Consumed by the Adequate and High-Salt Groups in the 2014–2018 National Health and Nutrition Survey, Japan
Source: Nutrients. 2021 Jul 28;13(8):2591. doi: 10.3390/nu13082591 (PMC8398318; doi:10.3390/nu13082591)
Supplement: Supplementary file 1 [file nutrients-13-02591-s001.zip › nutrients-1255018-supplementary.pdf]

Table S1. Standards for the "Healthy Meal".

**Dish type Ⅰ (Staple dish)**	**Dish type Ⅱ(Main dish)**	**Dish type Ⅲ (Side dish)**
A staple dish using unpolished rice or barley.<br>amounts to 40~70 g of carbohydrates<br>If the content of unrefined grains is high, intake should be limited to once a day.	amounts to 10~17g of protein from meat, fish, eggs, and soy products	Including two or more types of vegetables, potatoes, pulses (excluding soy), seaweeds, or mushrooms.<br>The amount of vegetables should be 100~200g

※ Energy. Energy of each dish should be less that 300kcal for type Ⅰ, less than 250kcal for typeⅡ, and less than 150kcal for type Ⅲ. When combining dish types Ⅰ~ Ⅲ at a meal, total energy should be less than 650kcal; ※ Salt. Salt content should be less than 1g per dish. When combining dish types Ⅰ~ Ⅲ at a meal, total salt should be less than 3g

Table S2. Criteria for categorizing dishes according to the definitions in the Japanese Food Guide Spinning Top.

**Type of dish**	**Criteria**	**Least amount of the corresponding food in the dish**	
Staple	amounts to 40g of carbohydrates	Rice, rice products	120g
		Bread (not including pastries)	80g
		Noodles, chinese noodles, pasta, other cereals	70g
Main	amounts to 6g of protein from meat, fush, eggs, and soy products	meat	50g
		fish and shellfish	50g
		eggs	50g
		soy products (including natto)	50g
		tofu	100g
Side	total amount of vegetables, potatoes, pulses (excluding soy), seaweeds, or mushrooms		70g
